# Color Doppler ultrasound diagnosis of intrarenal vein thrombosis: A rare case report and literature review

**DOI:** 10.1097/MD.0000000000010284

**Published:** 2018-03-30

**Authors:** Yixiu Zhang, Ying Wang, Jianchu Li, Sheng Cai

**Affiliations:** Department of Ultrasound, Peking Union Medical College Hospital, Chinese Academy of Medical Sciences and Peking Union Medical College, Beijing, China.

**Keywords:** Doppler, intrarenal vein thrombosis, ultrasound

## Abstract

**Rationale:**

We present a case of intrarenal vein thrombosis (IRVT) diagnosed by ultrasound (US). To the best of our knowledge, this is the first reported case in the imaging literature.

**Patient concerns:**

A 15-year-old boy with a 4-year history of thrombocytopenic purpura presented to the emergency room with a 2-day history of sudden-onset severe left flank pain associated with gross hematuria.

**Diagnoses:**

Hypercholesterolemia, proteinuria, and elevated plasma creatinine level were present. The US examination showed obscurely structured, sparsely distributed arterial and venous flow signals, and an increased resistance index (RI) in a localized area. The diagnosis was acute renal failure and nephrotic syndrome accompanied by left IRVT.

**Interventions:**

The patient was treated with anticoagulation therapy for 1 month.

**Outcomes:**

Clinical symptoms were relieved. The US re-examination revealed that the arterial flow spectra had returned to normal. Also, more venous flow signals were observed in the involved area, suggesting thrombolysis.

**Lessons:**

This previously unreported case should alert sonographers to include IRVT in the differential diagnosis of flank pain associated with hematuria. In such cases, both kidneys and different areas of the same kidney should be scanned and compared. Some features, including an obscure structure and an increased RI for the involved area indicate possible IRVT.

## 1 Introduction

Renal vein thrombosis (RVT) is defined as thrombus formation in the main renal vein or its intrarenal branches.^[1–3]^ The RVT could lead to a series of pathologic changes and clinical manifestations, including recurrent thromboembolic phenomena or renal failure. The RVT develops gradually, presumably beginning as a partial vein thrombosis,^[4,5]^ which would be a narrow but important window for early diagnosis of RVT. Ultrasound (US) is an important tool for evaluating RVT and its follow-up. Careful scanning can reveal even small changes caused by an intrarenal vein thrombosis (IRVT). Although thromboses have been reported in the main renal vein, detection of an IRVT has been rarely reported.

We describe an IRVT localized in only a portion of the involved kidney. The US findings, including those detected using gray-scale, color, and pulsed-wave Doppler, are comprehensively delineated.

## 2 Case report

A 15-year-old Chinese boy visited the emergency department for severe left flank pain of sudden onset accompanied by hematuria that occurred 2 days previously. He experienced significant tenderness, rebound tenderness, and percussion pain in the left renal area. The lower limbs were edematous. The boy had a 4-year history of thrombocytopenic purpura that was being treated with steroids. Laboratory findings revealed hypercholesterolemia (277 g/L), proteinuria (>3.0 g/L), and an elevated plasma creatinine level (2.79 mg/dL).

**Figure 1:**
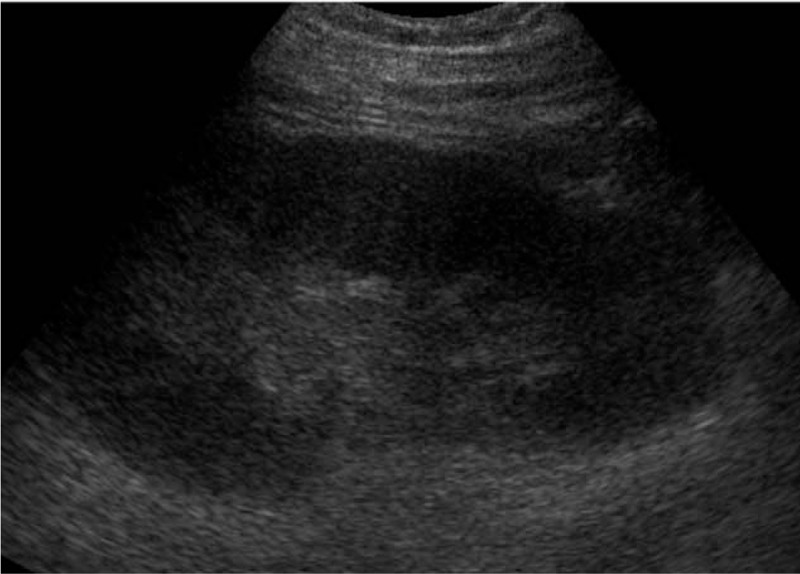
Upper pole of the left kidney was enlarged with an obscure structure.

**Figure 2:**
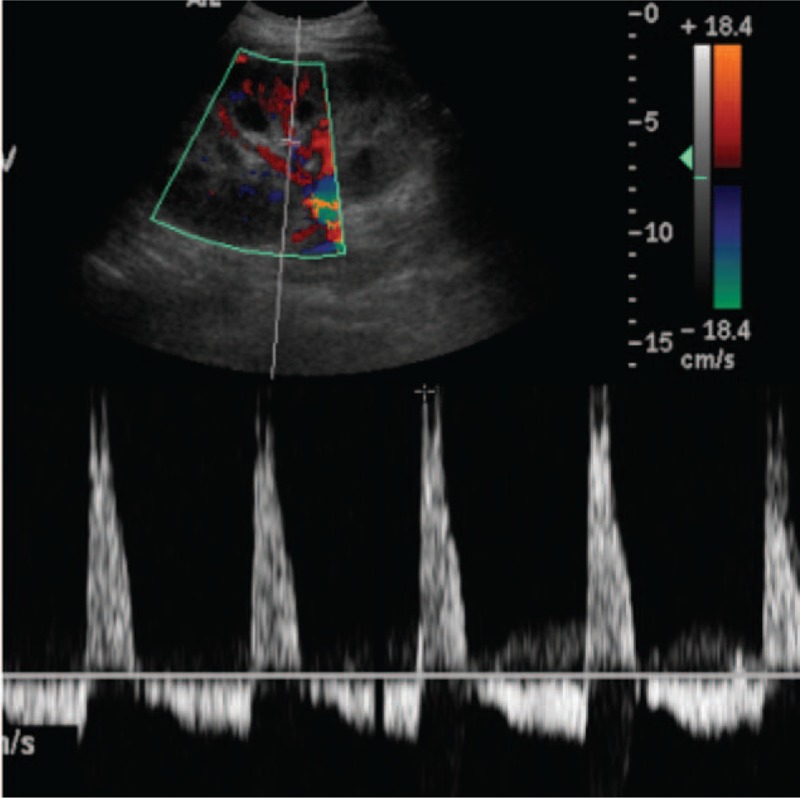
Sparsely distributed artery and venous flow signals were present. Reverse diastolic flow and an increased resistance index (RI; 0.83–1.00) were observed in this area.

**Figure 3:**
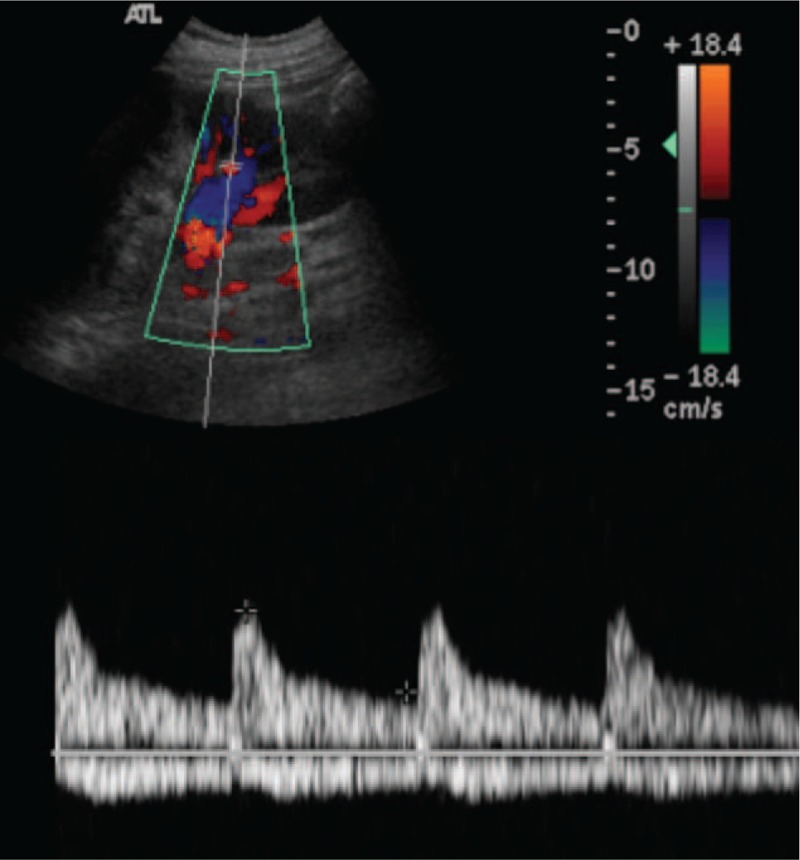
Normally distributed arterial and venous flow signals were present, and a normal resistance index (RI; 0.70–0.72) was observed in the lower pole of the same kidney.

Renal isotope scanning of the kidneys revealed poor parenchymal perfusion and minimal function of the left kidney, especially on the upper pole. A careful, thorough US examination showed, in gray-scale mode, that the upper pole of the left kidney was enlarged and obscure (Fig. 1). Further observation with color Doppler showed sparsely distributed arterial and venous flow signals in this area. Pulsed-wave Doppler showed different waveform changes in different parts of the same kidney. In the upper pole, there was reversed diastolic flow and an increased resistance index (RI; 0.83–1.00), whereas the lower part of the same kidney showed normal waveforms and a normal RI (0.70–0.72). To ensure a correct diagnosis, the RI was assessed for the segmental renal artery of the right kidney, which exhibited a normal RI (0.67; Figs. 2 and 3).

**Figure 4:**
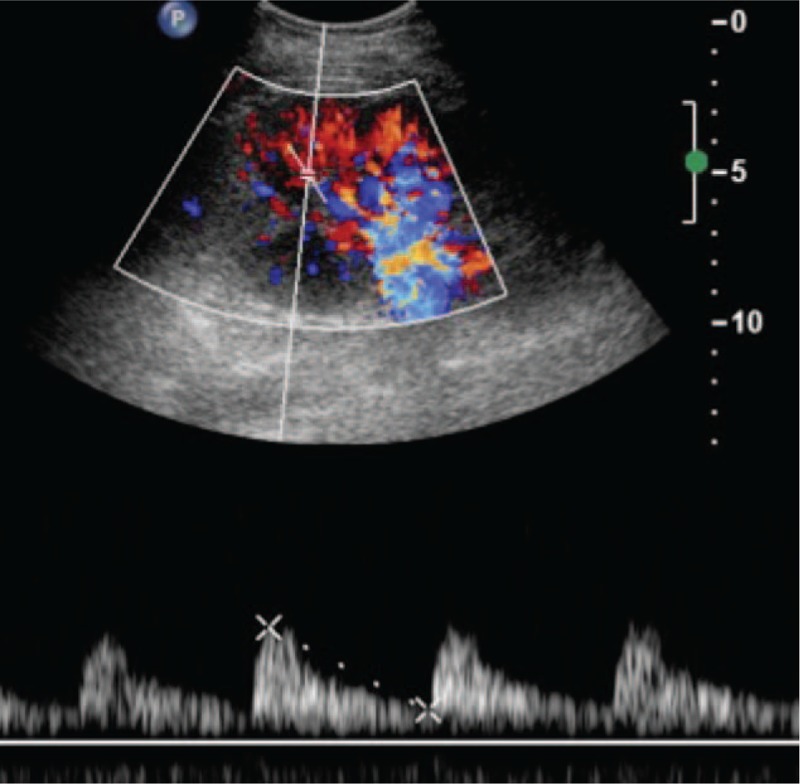
After treatment, increased arterial and venous flow signals were observed in the same area. Normal waveforms were present, and the segmental artery resistance index (RI) had returned to the normal range (0.6–0.72).

Considering all the above information, including the clinical manifestations and US examination results, we arrived at a diagnosis of acute renal failure, nephrotic syndrome, and IRVT of the left kidney. Immediate anticoagulation therapy with heparin and warfarin was prescribed. After 1 month of treatment, his clinical symptoms had disappeared completely, and renal function was improved. At the same time, US re-examination by the same radiologist showed that the left kidney was smaller, especially in the upper pole, than a month ago. Increased arterial and venous flow signals were observed in the same area. Normal waveforms were present, and the segmental arterial RI had returned to normal range (0.6–0.72; Fig. 4), suggesting thrombosis. Repeat renal isotope scanning showed increased parenchymal perfusion, rising from 12.1 to 19.9 mL/min, suggesting improved renal function. The patient signed informed written consent to report this case. The study was approved by the Institute Research Ethic Committee of Peking Union Medical College Hospital.

## 3 Discussion

The post-glomerular circulation is particularly prone to thrombosis because of its slow flow.^[4,5]^ A faulty coagulation mechanism and slow flow lays the groundwork for IRVT to occur, which was confirmed by histologic evidence provided by Searle et al^[6]^ and Nagra et al.^[7]^ Once a thrombus forms in the venous radicals, it gradually progresses to the main renal vein and vena cava.^[8,9]^ However, because of its indolent clinical course and inapparent imaging features at the early stages, it is difficult to diagnose it promptly and provide timely treatment. The detection rate of IRVT is low. Until now, no imaging studies of IRVT had been described in the literature.

In our case, the examination was timely because of the patient's obvious clinical symptoms. The enlarged, ambiguous structure of the kidney, sparsely distributed arterial and venous signals, and increased RI values were isolated to a localized area of the left kidney. Thus, a diagnosis of IRVT was highly suggested based on these US appearances combined with the classic “RVT diagnostic triad”: severe abdominal pain, gross hematuria, rapidly deteriorating renal function. The disappearance of venous flow signals in the involved area of the kidney might not be apparent because venous collaterals may quickly develop in RVT patients once thrombosis is complete.^[10–13]^

**Figure 5:**
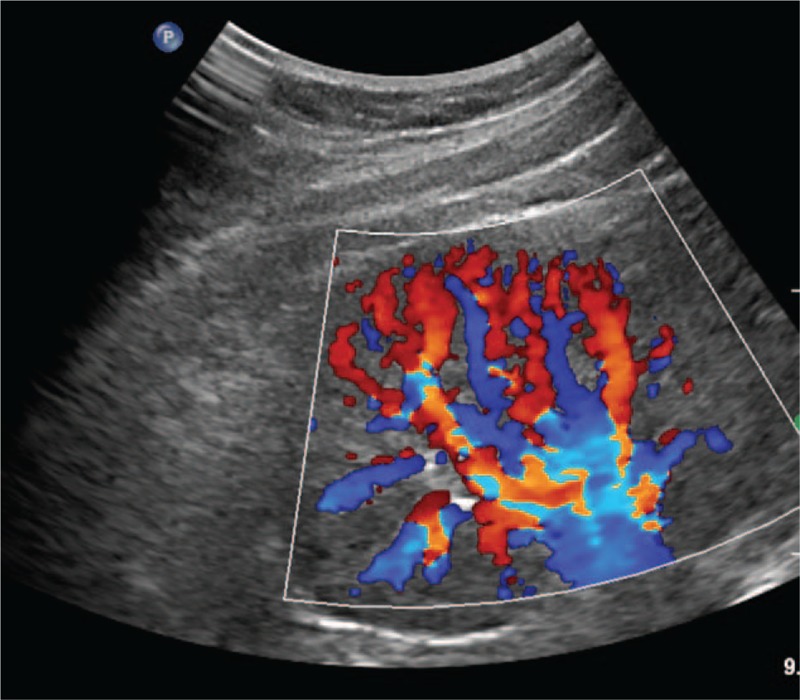
Vascular tree grows normally in the normal kidney. Both the main and arcuate arteries are easily visualized.

An increased RI is an important identification point for diagnosing IRVT, although it also occurs in other conditions. Hence, the general clinical information must be comprehensively analyzed. If the RI is increased in both kidneys, cardiac or kidney insufficiency should be considered. If an increased RI is present in a localized area of one side of a kidney, thrombus formation in small vessels could be the reason. In our case, the IRVT was formed in a small vein, which must be distinguished from renal tumors that have no obvious occupying effect. In the normal kidney, the vascular tree normally grows into other areas, and both the main artery and arcuate arteries are easily visualized (Fig. 5). As a tumor grows, small thrombus could be formed for tumor cells infiltration, causing an increased RI in a localized area.

**Figure 6:**
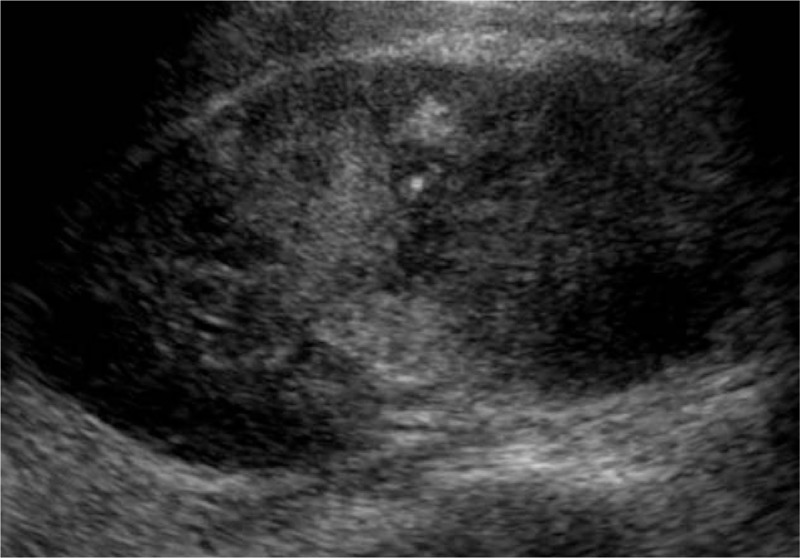
Local area structure was enlarged and obscure.

**Figure 7:**
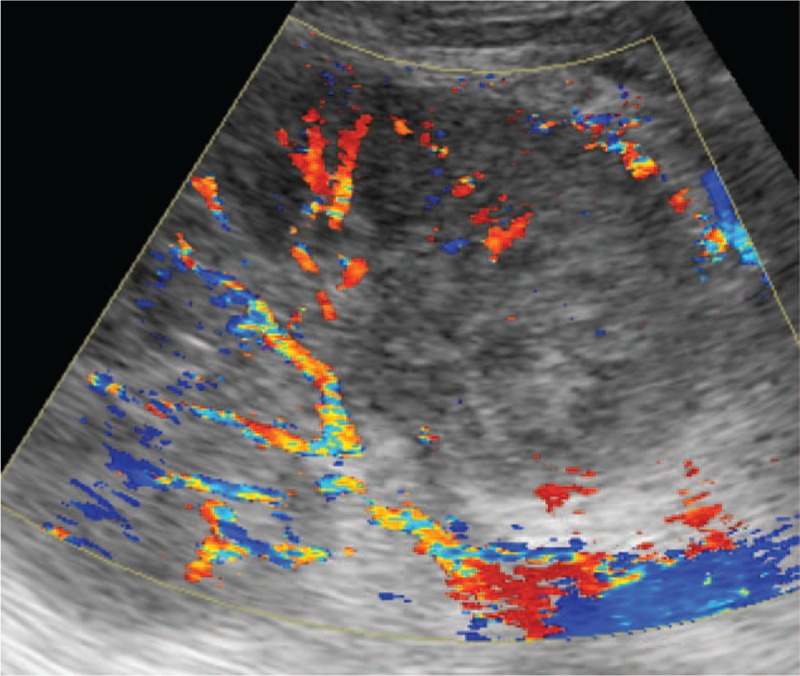
Neovascularization was quite thin, rigid, and discontinuous.

The US evaluation has been used in other renal transitional-cell carcinoma cases. As seen in Figure 6, it is similar to our case in gray-scale mode, with the lower pole of the kidney enlarged and obscure. Color Doppler, however, revealed that the morphology of the intraparenchymal arteries in the malignant case was quite different from that of the IRVT. Neovascularization was thin, rigid, and discontinuous (Fig. 7). Thus, the morphology of the intraparenchymal artery is important for differentiating IRVT from thrombus associated with malignant lesions.

## 4 Conclusion

There are still some difficulties associated in diagnosing IRVT by US. The primary step in diagnosis is comparative scanning of both kidneys and different areas of the same kidney. Features such as an obscure structure and an increased RI of the involved area should call for a high index of suspicion and early diagnosis of IRVT. Normal morphology of intraparenchymal arteries is important for differentiating IRVT from malignant lesions.

## Author contributions

**Conceptualization:** S. Cai.

**Data curation:** S. Cai.

**Formal analysis:** Y. Zhang.

**Funding acquisition:** J. Li.

**Investigation:** S. Cai.

**Writing – original draft:** Y. Wang, Y. Zhang.

**Writing – review & editing:** Y. Zhang.
